# Pancreatic cancer presenting with paraneoplastic thrombophlebitis–case report

**Published:** 2010-02-25

**Authors:** C Diaconu, D Mateescu, A Bălăceanu, M Marcu, V Jianu, A Stănică

**Affiliations:** * ‘Carol Davila’ University of Medicine and Pharmacy, Ilfov Clinical Hospital, Internal Medicine Department, BucharestRomania; ** Ilfov Clinical Hospital, Oncology Department, Bucharest Romania; *** ‘Carol Davila’ University of Medicine and Pharmacy, Ilfov Clinical Hospital, Anatomopathology DepartmentRomania; **** Ilfov Clinical Hospital, Computed Tomography Department, BucharestRomania

**Keywords:** tumor, gemcitabine, erlotinib

## Abstract

Context. The complex of symptoms in pancreatic cancer is vague, which often delays presentation and diagnosis. Thrombophlebitis is an unusual presentation of pancreatic cancer, which appears more frequent in the cancer of the body and tail of the pancreas.

Case report. We present a case of a 52–years–old woman who was admitted to the hospital for a superficial thrombophlebitis. At the abdomen ultrasound screening, multiple liver masses, relatively well defined, with a hypo–echoic center and a hyper–echoic periphery, were identified. The head of the pancreas was normal, the body and the tail could not be seen very well due to flatulence. After computed tomography, the diagnosis was ‘**Pancreatic tumor with multiple hepatic metastases (stage Ⅳ)**’. After the histopathological examination, 1,250mg/m^2^ of Gemcitabine was started on day 1 and 8 every 28 days, plus 100mg/day of Erlotinib (Tarceva), every day. At the end of the seventh month of treatment, the patient suffered an irreversible ischemic cardiac event.

Conclusion. Superficial thrombophlebitis can be the initial manifestation of the pancreatic cancer. Gemcitabine and erlotinib is now a FDA approved regimen for patients with metastatic pancreatic cancer. While the search for the best gemcitabine based backbone for advanced pancreatic cancer continues, studies of anti–angiogenic agents alone or in combination with traditional chemotherapy, should be undertaken, as they may improve overall survival in this group of poor prognosis patients.

## Introduction

The pancreatic cancer is the fourth leading cause of cancer death in North America, with the average age at diagnosis 60–65 years old [1]. The symptom complex is vague, which often delays presentation and diagnosis. Thrombophlebitis is an unusual presentation of pancreatic cancer, which appears more frequently in the cancer of the body and tail of the pancreas. Thrombophlebitis tends to occur in patients with more histological differentiated cancers. This is a relatively rare (5–15% of total presentations) presentation of pancreatic cancer; however, it is nonspecific and tends to occur more often in advanced disease.

There are few long–term survivors and poor response to combined modality treatment. The 5– year survival is under 2%. The majority (80%) of patients present with an unresectable disease [1]. The epidermal growth factor receptor (EGFR) tyrosine kinase inhibitors may be beneficial combined with chemotherapy, in these patients.

## Case report

**Fig 1:**
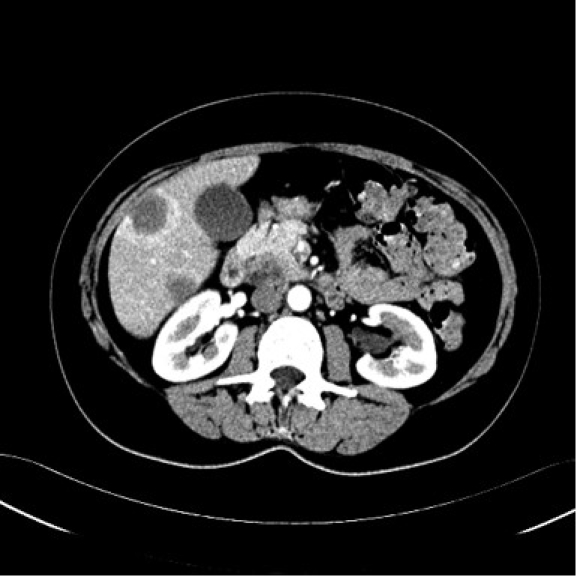
Abdominal computed tomography scan with contrast during the arterial phase. Multiple low– density masses in the liver;ill defined,  low–density mass, in the pancreas.

**Fig 2:**
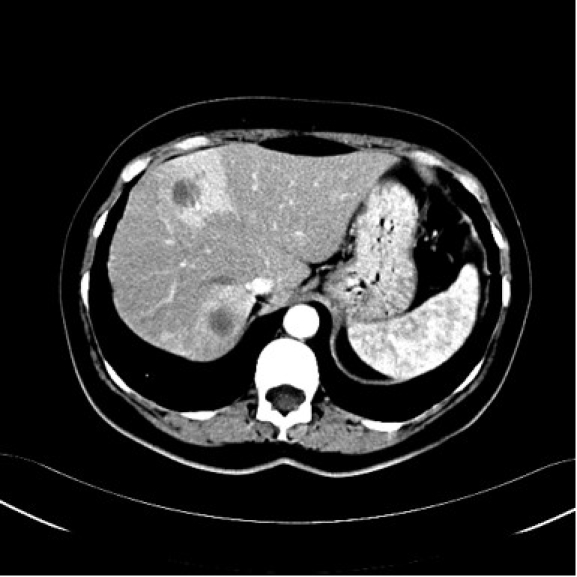
Abdominal computed tomography scan with contrast during the arterial phase. Multiple round– oval hypodense structures, with hyperdense periphery, in the liver.

**Fig 3:**
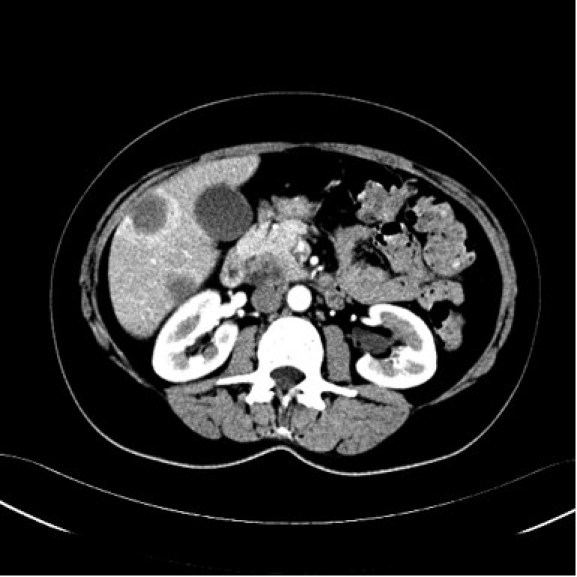
Abdominal computed tomography scan with contrast during the arterial phase. Multiple low– density masses in the liver; ill defined, low–density mass, in the pancreas.

**Fig 4:**
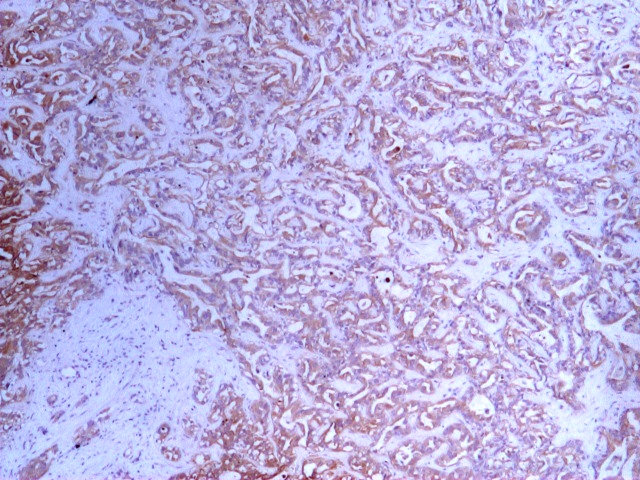
Hepatic metastatic lesion biopsy. Immunohistochemistry, CK 7 (plus), 4x.

**Fig 5:**
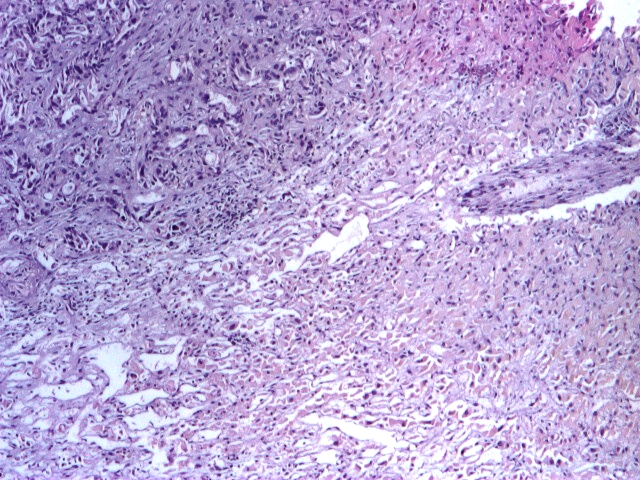
Hepatic metastatic lesion biopsy. There are some little malignant glands in the left quadrant – pancreatic adenocarcinoma metastasis. H and E stain, 4x.

**Fig 6:**
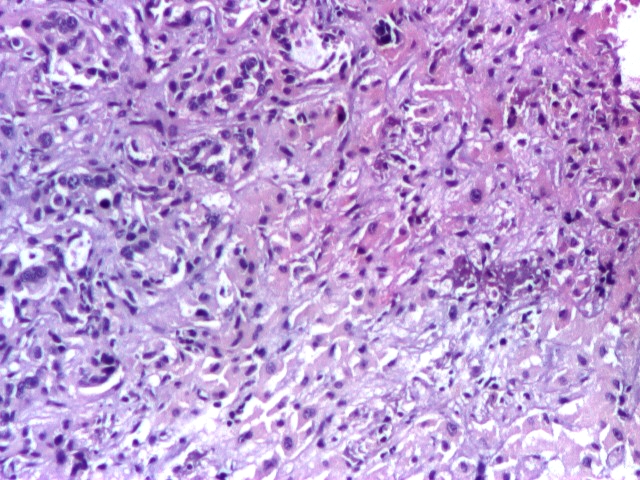
Hepatic metastatic lesion biopsy. There are some little malignant glands in the left quadrant – pancreatic adenocarcinoma metastasis. H and E stain, 10x.

A 52–year–old Caucasian woman was admitted to the Internal Medicine Department with a 1– week history of edema, erythema and pain of the left lower extremity, diagnosed as superficial thrombophlebitis. The physical exam was normal, except for the thrombophlebitis. She had no personal pathological history, only a brother with malignant lymphoma. At the ultrasound screening of the abdomen multiple liver masses were identified. They were relatively well defined, with a hypo–echoic center and a hyper–echoic periphery, the largest measuring 45mm. The head of the pancreas was normal, the body and the tail could not be seen very well due to flatulence. Routine blood chemistry including liver and pancreatic function tests and blood cell count were normal. Computed tomography scan revealed multiple low–density masses measuring between 20–47 mm in the liver; a low–density ill–defined mass of 27/23mm, in the pancreas, with involvement of the uncinate process (Fig 1, Fig 2, Fig 3). The diagnosis was **Pancreatic tumor with multiple hepatic metastases (stage Ⅳ)**. The patient was referred to the Oncology and then Surgery Department, where she underwent laparoscopy with biopsy of a secondary hepatic lesion. The histopathological and immunohistochemical result was ‘liver biopsy fragment with neoplastic infiltration of well–differentiated adenocarcinoma, citokeratin 7 and CA 125 positive in tumor cells, citokeratin 20 and 19 negative in tumor cells, OCH1E5 positive in the normal hepatocytes, negative in tumor cells, CDX2 negative, synaptophysin negative, chromogranin negative’ (Fig 4, Fig 5,Fig 6).

Conclusion: the histopathological aspect correlated with the immunohistochemical tests supports the diagnosis of adenocarcinoma with the point of departure in the epithelium of biliopancreatic ducts.

1,250mg/m^2^ of Gemcitabine was started on day 1 and 8 every 28 days, plus 100mg/day of Erlotinib (Tarceva) every day. Erlotinib is a small–molecule tyrosine kinase inhibitor of epidermal growth factor receptor (EGFR). At the beginning of the treatment, the patient had Hb of 9.6g/dL, platelets of 280,000/mm^3^ and white blood cells of 7,000/mm^3^. The body surface was of 1.59 m^2 ^.

The patient developed recurrent episodes of migratory superficial thrombophlebitis, treated with heparin,' then oral anticoagulants during the whole course of the disease.

After the second month of treatment, the patient became icteric, so she was administered 1,025U/L of gammaglutamiltranspeptidase and 311U/L of alkaline phosphatase. She underwent a palliative biliary drainage. After 3 weeks of Erlotinib therapy, she developed an acneiform eruption on the face and chest (anterior and posterior). A therapeutic protocol was applied for the side effects of tirosinkinasis inhibitors: 1,000mg/day of Azitrox for 7 days, local applications of 3% Boric acid solution and Zineryt solution for 7 days [2,3]. In the second phase the patient received Diprogenta cream for 10 days, then Ruboril for the inflammatory erythema.

The patient became anemic and received transfusions of erythrocytes concentrate repeatedly. The white blood cells and platelets were normal.

After 4 months of treatment, Gemcitabine was stopped because the medullar toxicity occurred and the patient was treated only with Tarceva. Her evolution was good, only with mild pain in the upper abdomen (the only analgesic needed was dihydrocodein). At the end of the seventh month of treatment, the patient suffered an irreversible ischemic cardiac event.

## Discussion

Gemcitabine is the first line therapy for patients with metastatic pancreatic adenocarcinoma with a median overall survival of 5.65 months, progression free survival of 9 weeks and a clinical benefit response of 23.8% [4]. Our patient had prolonged survival of 27 weeks that may be attributable to the addition of erlotinib. Erlotinib (Tarceva) is a human epidermal growth factor receptor type 1/epidermal growth factor receptor (HER1/EGFR) tyrosine kinase inhibitor initially approved by the US Food and Drug Administration in the treatment of patients with locally advanced or metastatic cancer. Erlotinib is the only oral epidermal growth factor tyrosine kinase inhibitor that has been shown to prolong overall survival in patients with advanced pancreatic cancer, although the benefit appears to be modest. It was approved by the Food and Drug Administration (FDA) in 2005, in combination with gemcitabine in the treatment of patients with locally advanced, unresectable or metastatic pancreatic carcinoma, who have not received previous chemotherapy [5,6].

The most common adverse reactions in patients with pancreatic cancer receiving 100 mg of Tarceva plus Gemcitabine are fatigue, nausea, anorexia and diarrhea. Our patient developed only the acneiform rash. In terms of clinical predictors of response, the development of skin rash has been suggested as a surrogate marker of favorable response to EGFR inhibition. The characteristic of EGFR inhibitor rash is papulopustular, predominantly occurring on the face and upper torso within two weeks from the start of treatment. Although the etiology remains unclear, the rash is postulated to occur secondary to EGFR inhibition in the skin [2]. Optimal dosage of erlotinib in advanced pancreatic cancer patients also remains at question.  In vitro studies suggest that drug concentration directly correlates with degree of antitumor effect, however, underscoring the importance of adequate dosing. Furthermore, the higher the doses of EGFR inhibitor are, the greater the incidence of rash [3]. Regarding the data suggesting that rash may be a biomarker for the tumor's response to EGFR inhibition, this dose– toxicity relationship suggests the need for future study of dose escalation for the development of the rash. It is unknown whether higher doses of erlotinib can induce rash in a greater proportion of patients, and whether this would result in improved clinical outcomes without unacceptable toxicity. Given that even mild–to–moderate skin rash can result in significant patient distress and impairment, the clinical benefit of anti–EGFR therapy must be carefully weighed against the toxicity of this common side effect.

Clinical trials involving other tyrosine kinase inhibitors and anti–angiogenic agents in pancreatic cancer are currently ongoing. The results of these and other ongoing clinical trials will help us redefine this regimen and determine the optimum doses and cycle length, for the most favorable results with minimum toxicities.
